# Evolutionary Changes of *GT1* Provide Insights Into the Adaptation of Butterflies to Plant Feeding

**DOI:** 10.1002/ece3.72291

**Published:** 2025-11-05

**Authors:** Jinyu Wu, Hengyu Yan, Wanjiang Tang, Zhengyang Li, Amrita Chakraborty, Zhengbo He, Cao Zhou, Shulin He

**Affiliations:** ^1^ College of Life Sciences Chongqing Normal University Chongqing China; ^2^ Chongqing Key Laboratory of Vector Control and Utilization, Institute of Entomology and Molecular Biology Chongqing Normal University Chongqing China; ^3^ Faculty of Forestry and Wood Sciences Czech University of Life Sciences Prague Prague Czechia

**Keywords:** adaptive evolution, butterfly, gene duplication and loss, glycosyltransferase 1

## Abstract

*Glycosyltransferase 1* (*GT1*) genes are involved in insect detoxification, olfactory perception, and endogenous metabolic regulation, and they play a role in the adaptation of butterflies to their specific feeding habits. However, their evolutionary trajectories in butterflies remain elusive. To reveal the evolutionary histories of the *GT1* gene family and its relationship with feeding niches in butterflies, we systematically identified *GT1* genes across 69 butterfly genomes and inferred their duplication and loss events by reconciling the gene tree with a species tree. Subsequently, the duplication modes and collinearity of these genes were determined between four selected species, followed by tests of selection pressures on the sites and branches of *GT1* genes. In addition, the expression patterns of different *GT1* gene subfamilies in the larval gut and adult antenna of two representative species were compared. Our results revealed substantial variation in the *GT1* gene numbers among butterfly species, but no significant difference between generalists and specialists. Most *GT1* genes originated via tandem duplications and are located within collinear blocks. Among the 13 subfamilies, the most expanded subfamilies, *UGT33* and *UGT40*, harbored multiple positively selected sites, suggesting functional diversification. In addition, most *GT1* genes expressed in adult antennae differ from those in larval guts, suggesting a divergence between female host selection and larval feeding preferences. Overall, the positive selection of specific sites and gene expression, not gene numbers or expansions, in the *GT1* gene family is likely involved in the adaptation of plant utilization in butterflies. This study provides novel insights into the evolutionary mechanisms of detoxification‐related gene families in insects and enhances our understanding of how butterflies adapt to chemical challenges posed by host plants.

## Graphical abstract

We investigated the evolution of glycosyltransferase 1 (GT1) genes in 69 butterfly species to explore their association with host plant adaptation. Our results reveal frequent tandem duplications and adaptive selection in GT1s, especially in UGT33 and UGT40 subfamilies, alongside expression patterns suggesting functional diversification.

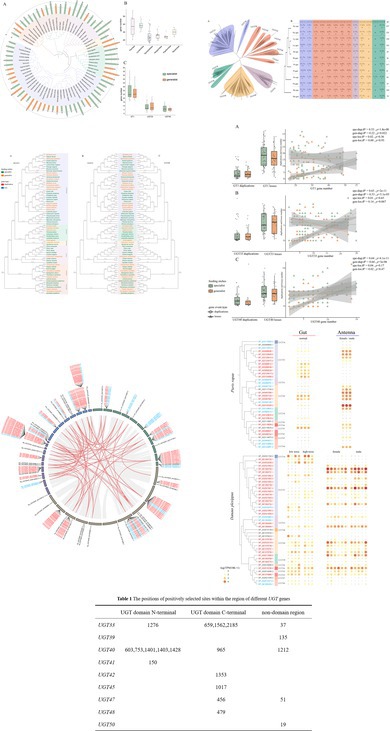

## 1 Introduction

The chemical interactions between herbivorous insects and their host plants are a classic model for studying adaptive evolution and coevolutionary processes. Plants have evolved diverse specialized metabolites, including alkaloids, glycosides, and terpenoids, as part of sophisticated chemical defense systems to deter herbivory. Insects, in turn, have developed a range of behavioral and physiological counter adaptations, such as selective oviposition and metabolic detoxification (Ehrlich and Raven 1964), which have driven lineage‐specific expansions and functional diversification of relevant gene families (Nallu et al. 2018). Among herbivorous insects, Lepidoptera, particularly butterflies, have emerged as important systems for investigating plant–insect coevolution due to their diversified host‐use strategies and specialized chemosensory adaptations (Brady et al. 2021).

The butterfly adults and larvae developed distinct strategies for plant utilization. Adults rely on antennal chemosensation to locate or select suitable oviposition host plants (Futuyma and Moreno 1988; Renwick and Lopez 1999), whereas larvae depend on detoxification genes in the gut to process ingested plant chemicals (Yu et al. 2016). Host selection is typically performed by adult females during oviposition and influenced by volatile and contact‐based chemical cues emitted by plants (Haribal and Renwick 1996, 1998; Reudler Talsma et al. 2008). For example, *Melitaea cinxia* females exhibited oviposition preference through contact chemoreception by selecting host plants rich in iridoid glycosides; similarly, the antennal responses of *Papilio* butterflies to different plant volatiles are closely linked to their oviposition choices (Inoue et al. 2023). Larval performance is subsequently constrained by the nutritional quality and chemical defenses of the selected host plants; preferred hosts would accelerate larval development and growth (Reudler Talsma et al. 2008). In some taxa, larvae not only tolerate toxic plant metabolites but also sequester them for their defense (Nishida 2002). For instance, *Junonia coenia* can feed on hosts containing iridoid glycosides and partially sequester them for defense (Richards et al. 2012); Heliconiini species can sequester cyanogenic glucosides and alkaloids from *Passiflora* plants for defense (Castro 2018).

Regarding larval feeding, butterflies can range from highly specialized in a single host plant to generalist across multiple host plant families, and this difference in diet breadth is assumed to be closely associated with their detoxification capabilities (Kawahara et al. 2023). For example, *Danaus plexippus* feeds almost exclusively on plants of *Asclepias*, particularly various species of milkweed containing cardenolides (Greenstein et al. 2022); *Heliconius melpomene* primarily utilizes *Passiflora* containing cyanogenic glucosides (Greenstein et al. 2022). In contrast, *Vanessa* can feed on the leaves of three host plants characterized by distinct specialized metabolites: willowherb (Epilobium; Onagraceae), common plantain ( *Plantago lanceolata* ; Plantaginaceae), and oxeye daisy ( *Leucanthemum vulgare* ; Asteraceae) (Gallon and Smilanich 2023). However, even generalists are not free to exploit any host at random: Shifts tend to occur among chemically similar plants, indicating that plant specialized metabolite structure acts as a filter on host use evolution (van der Linden et al. 2021). The diversification of butterfly host use is both driven by and contributes to gene and genome duplications, particularly in detoxification‐related gene families that enable adaptation to chemically diverse plants (Edger et al. 2015; Allio et al. 2021), such as carboxylesterases and glutathione‐S‐transferases (Breeschoten et al. 2022).

Insects often employ a three‐phase system to metabolize these plant‐derived compounds: detoxification, conjugation, and excretion (Després et al. 2007). As key Phase II enzymes, members of the *glycosyltransferase 1* (*GT1*) gene family play multifunctional roles in mediating insect–plant interactions (He et al. 2017; Teze et al. 2022). In addition to basic cellular functions such as glycosylating cell membrane components (Nagare et al. 2021), it can catalyze the glycosylation of plant specialized lipophilic or unstable metabolites to water‐soluble stable products, which effectively neutralizes the toxic metabolites by increased solubility and facilitated excretion (Yonekura‐Sakakibara and Hanada 2011; Teze et al. 2022). Furthermore, *GT1* genes are involved in olfactory and endogenous metabolic processes. Previous studies have demonstrated that gene duplication and loss in insect *GT1* families are closely associated with host shifts (Rane et al. 2016; Wu et al. 2024). Notably, *GT1* genes in Lepidoptera undergo frequent duplication and loss events, exhibiting highly dynamic lineage turnover, with their evolution closely associated with adaptation to angiosperm host plants (Wu et al. 2024). The complexity of host plant utilization strategies in butterflies may be influenced by the evolution of the detoxification enzyme *GT1* and its expression patterns in host plant usage‐related tissues, gut, and antennae. While direct evidence for such a role in butterflies remains scarce, studies in other Lepidoptera have shown that *UDP‐glycosyltransferases* can act as key determinants of host plant range in the *Spodoptera* specialists and generalists (Wang et al. 2024). Moreover, host plant adaptation in Lepidoptera has been shaped by multiple, independent evolutionary events (Luo et al. 2025), providing repeated diversification of detoxification gene families such as *GT1*. However, systematic investigations on the *GT1* gene evolutionary characteristics in butterfly species are still limited.

Based on our previous broad analyses of the *GT1* gene family across insects, this study focused on 69 herbivorous butterfly species, systematically identified *GT1* family members, investigated their evolutionary dynamics, and assessed related gene family expansions and signals of positive selection. By integrating transcriptomic data from larval guts and adult antennae, we revealed the expression patterns of *GT1* subfamilies associated with oviposition preference and larval feeding behavior. Our results reveal that the evolutionary dynamics of certain *GT1* genes have facilitated adaptive responses to host utilization and indicate the patterns of butterfly adaptation to host plants.

## 2 Materials and Methods

### 2.1 Data Collection

Genomic data of butterfly species were downloaded from NCBI (accessed on January 20, 2025). Genome assembly quality was assessed using BUSCO v5.4.3 with the “*insecta_odb10”* database; genomes with a complete BUSCO score below 95% were considered low‐quality assemblies and excluded from subsequent analyses (Waterhouse et al. 2018). A total of 69 lepidopteran genomes were selected, including 15 species from the RefSeq database and 54 species from the GenBank database (Table S1). In addition, *UGT* protein sequences from six moth species (Z*ygaena filipendulae*, *Helicoverpa armigera*, *Bombyx mori* , *Plutella xylostella*, *Spodoptera frugiperda* , and *Plutella xylostella*) were downloaded from the *UGT* classification database and used as references to aid in the accurate classification of *GT1* subfamilies.

Data on butterfly feeding niches were obtained from a previously published study (Kawahara et al. 2023). In the present study, we did not account for the specific composition or concentration of specialized metabolites in host plants. Instead, species were classified based solely on the taxonomic breadth of their host range. Species that feed exclusively on host plants within a single plant family were classified as specialists, whereas those that feed on host plants from multiple families were classified as generalists (Table S1).

### 2.2 *GT1* Gene Identification

We identified *GT1* genes separately for reference and nonreference genomes. For reference genomes retrieved from the RefSeq database, we first extracted the longest isoform of each gene based on the annotation file using the orthologr package (Drost et al. 2015). The corresponding protein sequences were then subjected to *GT1* annotation using run_dbcan, which integrates HMMER, Diamond, and dbCAN_sub (Zheng et al. 2023). The annotation of *GT1s* was considered reliable only when all three tools produced the same prediction. Subsequently, the predicted *GT1* protein sequences were clustered using CD‐HIT v4.8.1 to generate a non‐redundant *GT1* protein database for nonreference identification analysis (Li and Godzik 2006). For genomes retrieved from the GenBank database, *GT1* genes were first predicted by homology‐based search using genblastG against the constructed nonredundant *GT1* protein database. Gene models lacking either start or stop codons were filtered out using GffRead v0.12.7, and redundant sequences were also removed (Pertea and Pertea 2020). In cases when *GT1* gene models overlapped at the same genomic locus, only the one with the highest genblastG alignment score was retained. The coding and protein sequences of the retained gene models were then reannotated by run_dbcan, following the same criteria as for reference genomes, and only those predicted by all three methods were considered high‐confidence *GT1* members.

### 2.3 Phylogenetic Analysis of the *GT1* Gene Family

To infer the phylogenetic relationships of *GT1* genes in butterflies, we aligned the *GT1* protein sequences using both MAFFT v7.520 and MUSCLE v5.1 (Katoh and Standley 2013; Edgar 2004). The alignments were then refined using RASCAL v1.34, and the original and refined alignments were evaluated with normd v1.2 (Thompson et al. 2003). The alignment with the highest score was selected for subsequent phylogenetic inference by maximum likelihood using IQ‐TREE v2.1.3. The resulting *GT1* gene tree was visualized using Dendropscope (Nguyen et al. 2015). Finally, the total number of *GT1* genes and the numbers of *UGT33* and *UGT40* subfamilies across species with different feeding habits were visualized using bar plots.

### 2.4 Gene Duplication and Loss Inference in Butterflies

To explore *GT1* gene copy number variation during butterfly evolution, we reconciled the *GT1* gene tree with the species tree using Notung v2.9.1.5 (Durand et al. 2006). The species tree from a previous study (Kawahara et al. 2023) was pruned to include only butterfly species of interest using the keep.tip function from the ape R package (Paradis and Schliep 2019). The reconciliation analysis was performed with the following parameters: gene loss cost = 1.0, duplication cost = 1.5, horizontal transfer cost = 3.0, codivergence cost = 0, and edge weight threshold = 90% of the maximum edge weight.

### 2.5 Duplicate Mode Inference and Collinearity Analysis

To represent phylogenetic and ecological diversity, we selected four butterfly species from the RefSeq database, with each representing a different family and covering both generalist and specialist feeding types: *Aricia agestis* (generalist, Lycaenidae), *Danaus plexippus* (specialist, Nymphalidae), *Papilio machaon* (generalist, Papilionidae), and *Pieris rapae* (specialist, Pieridae) for duplication and collinearity analyses. The duplication modes of *GT1* genes in each species were inferred using the duplicate_gene_classifier script from MCScanX (Wang et al. 2012). Collinearity analysis was performed for four representative butterfly species (Aa, *A. agestis*; Pr, *P. rapae* ; Pm, *P. machaon* ; Dp, *D. plexippus* ) using MCScanX, and the results were visualized by Circos implemented in TBtools v2.119 (Chen et al. 2023).

### 2.6 Selection Pressure Analysis of *GT1* Genes

Selection pressure analysis of *GT1* genes was conducted using HyPhy v2.5.62. Protein sequence alignments were converted into codon alignments using PAL2NAL v14 (Suyama et al. 2006). Site‐specific selection pressures were determined using FEL (Pond and Frost 2005) and MEME (Murrell et al. 2012) models to detect signals of pervasive and episodic positive selection across *GT1* coding sequences. Sites were considered positively selected when they were under positive selection with *p* < 0.05 inferred in both FEL and MEME models.

### 2.7 Expression Profiling of *GT1* Genes

To exclude potential confounding effects, we only selected samples neither infected with pathogens nor treated with chemicals. The larval gut and adult antenna of *D. plexippus* (SRA Project No.: PRJNA382538; PRJNA961744) and *P. rapae* (SRA Project No.: PRJNA554441; PRJNA631907) were filtered using Fastp v0.23.2 (Chen 2023) to obtain high‐quality data. The filtered reads were then mapped to the reference genomes using HISAT2 v2.2.1 (Kim et al. 2019); mapped reads were processed with SAMtools v1.16 and FeatureCounts v2.0.6 to construct gene expression matrices (Danecek et al. 2021; Liao et al. 2014). Raw expression values were transformed into transcripts per million 10 K (TPM10K) as the product of TPM and the total number of genes in the reference transcriptome (Munro et al. 2022). This normalization corrects for variation in reference gene set size across species and thereby reduces biases in cross‐species comparisons. Subsequently, normalized expression levels were log‐transformed for visualization.

### 2.8 Statistical Analysis

To assess the correlation between the number of *GT1* genes and the evolutionary trajectories of butterfly specialists and generalists, a time‐calibrated species tree with divergence times was used (Kawahara et al. 2023). The *phylANOVA* function in the phytools package was applied to test for statistical differences in the total number of *GT1*, *UGT33*, and *UGT40* genes among species with different host plant preferences (Rohlfs and Nielsen 2015). In addition, *ANOVA* was performed to determine the statistical differences in the *GT1* gene numbers among butterfly families.

## 3 Results

### 3.1 High Intra‐Lineage Variation in *GT1* Copy Number

**FIGURE 1:**
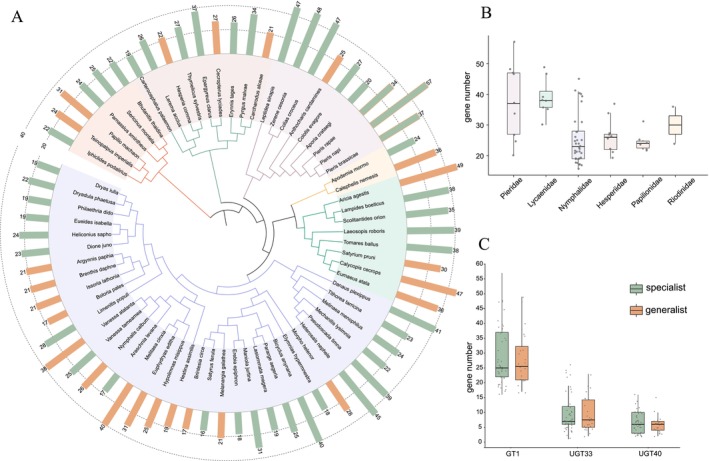
Phylogenetic tree of 69 butterfly species visualized using iTOL (A). Inner branches and colors indicate family‐level taxonomy (Kawahara et al. 2023), while outer bar plots represent the copy number of *GT1* genes. Orange and green bars denote generalist and specialist species, respectively. The total *GT1* gene numbers and the numbers of *UGT33* and *UGT40* subfamilies between generalist and specialist butterfly species (B). The gene number of *GT1*, *UGT33*, and *UGT40* subfamilies in butterfly families (C).

The number of *GT1* genes varies substantially among butterfly species, with an average of 29 gene copies. The highest number of *GT1* genes (57) was found in *P. napi* , while the lowest (16) was found in *Satyrus ferula* (Figure 1A). At the family level, the average number of *GT1* genes differs substantially (F_5‐63_ = 6.70, *p* = 4.58e‐05); 25 in Papilionidae and Nymphalidae, 27 in Hesperiidae, 30 in Riodinidae, 38 in Pieridae, and 39 in Lycaenidae. *GT1* gene numbers were generally lower in Nymphalidae, but a few species exhibited large numbers, such as 45 in *Heterosais nephele* and 41 in *D. plexippus* ; whereas Lycaenidae generally harbors higher *GT1* numbers. Hesperiidae and Papilionidae displayed relatively stable gene numbers; in contrast, Pieridae exhibited the greatest interspecific variation, ranging from 20 to 57. In addition to the variation observed across families, we also detected considerable differences in *GT1* gene numbers within genera. For example, species within the genus *Pieris* differ by 20 *GT1* gene copies, indicating that *GT1* gene expansion or contraction may occur among closely related species. However, the average copy numbers of *GT1* genes showed no obvious differences in gene counts between generalist and specialist butterflies (Figure 1C). To further investigate the relationship between *GT1* gene copy number and dietary breadth (generalist vs. specialist) in butterflies, we performed *phylANOVA* analyses incorporating the butterfly phylogenetic framework. Specifically, we tested for differences in the total number of *GT1* genes between generalist and specialist species and found no significant difference in total *GT1* (F_1‐67_ = 0.19, *p* = 0.71).

### 3.2 Divergent Expansion of UGT Subfamilies Across Butterfly Families

**FIGURE 2:**
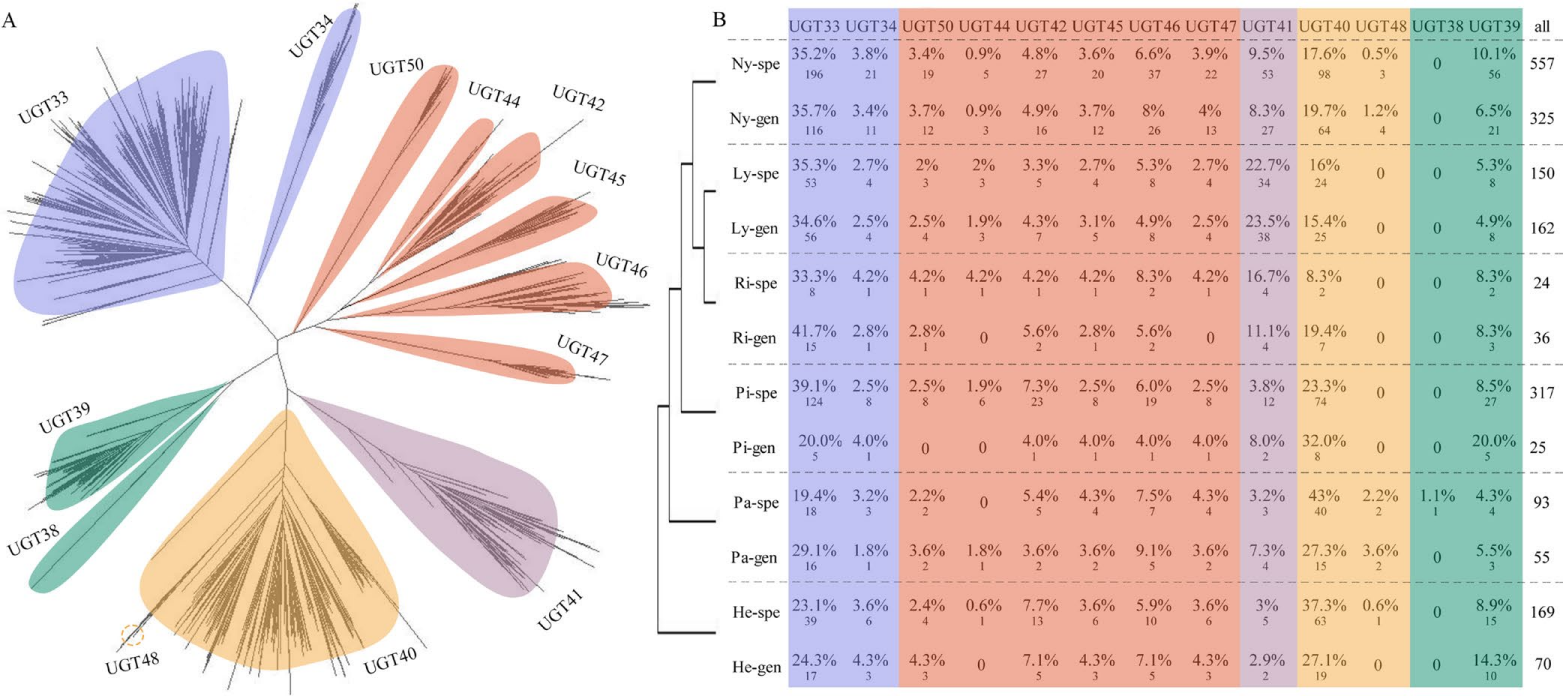
Phylogenetic tree of *GT1* genes from butterflies and *UGT* genes from other Lepidopteran species constructed using IQtree2 and visualized with Dendroscope (A). Based on *UGT* classification data and *GT1* clade topology, butterfly *GT1* genes were grouped into distinct subfamilies. To further investigate the distribution patterns of *UGT* gene lineages among butterfly taxa, we classified species into 12 groups based on their feeding type (generalist [gen] or specialist [spe]) and family affiliation (Nymphalidae [Ny], Lycaenidae [Ly], Riodinidae [Ri], Pieridae [Pi], Papilionidae [Pa], and Hesperiidae [He]) (B). For each of these 12 phylogenetic‐feeding groups (family × feeding type), we calculated the proportion of genes falling into each *UGT* phylogenetic clade to assess potential associations between specific *UGT* sublineages and butterfly ecological traits.

By constructing a phylogenetic tree that integrates *GT1* gene sequences from butterflies and *UGT* sequences from a few studied moth species, we classified butterfly *GT1* genes into distinct clades based on the tree topology and clustering patterns of different *UGT* subfamilies (Figure 2A). Overall, the *GT1* genes could be divided into four major clades, each of which contained several subclades. *UGT33* and *UGT40* were the predominant *GT1* subfamilies in butterflies, constituting about 55% of the *GT1* gene numbers in each species. In contrast, *UGT34*, *UGT38*, *UGT44*, *UGT45*, *UGT47*, *UGT48*, and *UGT50* exhibited relatively low copy numbers, and some of these subfamilies were completely absent in certain species.

Considerable differences were also observed in *UGT* subfamily composition across butterfly families. Nymphalidae (Ny), Lycaenidae (Ly), Riodinidae (Ri), and Pieridae (Pi) generally harbor more *UGT33* copies (33.3%–41.7%) but fewer *UGT40* copies (8.3%–26.8%) than Hasperidae and Papillionidae (27.1%–43% UGT40; 19.4%–29.1% UGT33); within the generalist Pieridae category, which is represented by only a single species, the *UGT33* copy number is lower (20%) while the *UGT40* copy number is higher (32%) compared with the other families. Additionally, the *UGT41* subfamily is more expanded in Lycaenidae (Ly) than in other families. Despite these taxonomic differences, no clear difference was detected between generalist and specialist species in terms of *GT1* gene distribution across different *UGT* subfamilies (Figure 2B), including in the large subfamilies *UGT33* (phylANOVA, F_1‐67_ = 0.052, *p* = 0.86) and *UGT40* (phylANOVA, F_1‐67_ = 1.29, *p* = 0.34).

### 3.3 Frequent GT1 Gene Turnover Independent of Host Range

**FIGURE 3:**
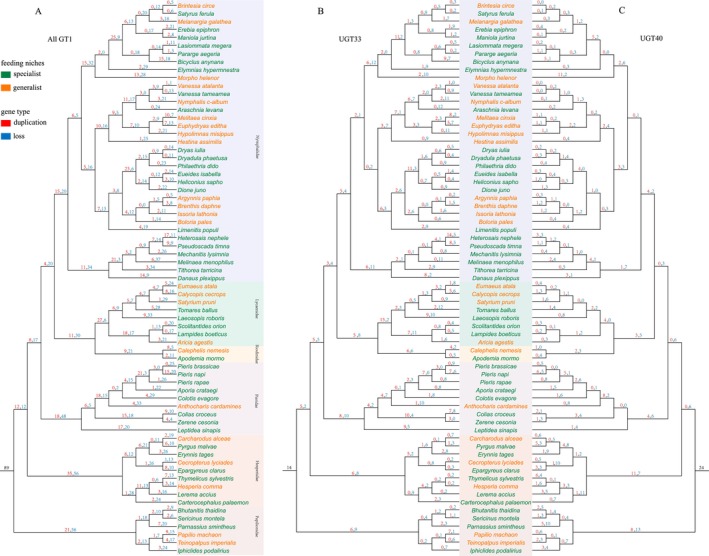
Duplication and loss events of *GT1* (A), *UGT33* (B), and *UGT40* (C) genes in butterflies. Red numbers above branches indicate gene duplications, blue numbers for gene losses, and the black number at the root node for the ancestral *GT1* gene copy number.

By reconciling the *GT1* gene phylogeny with the phylogenetic tree of butterflies, we inferred the gene duplication and loss events during butterfly evolution (Figure 3). Our analysis revealed that the common ancestor of butterflies possessed a large number (89) of *GT1* gene copies. Throughout butterfly diversification, *GT1* genes underwent extensive duplications and losses, particularly during the divergence of major butterfly families. Substantial changes in *GT1* gene copy numbers were observed in the lineages leading to *Papilionidae* (Duplications: 21, Losses: 56) and *Hesperiidae* (Duplications: 35, Losses: 56). For specific subfamilies, the ancestral gene number was estimated to be 14 for *UGT33* and 24 for *UGT40*. Gene turnover also occurred frequently during species divergence, for example, in *Morpho helenor* (*UGT33*:2,10; *UGT40*:11,2) and *Laeosopis roboris* (*UGT33*:9,10; *UGT40*:0,8).

**FIGURE 4:**
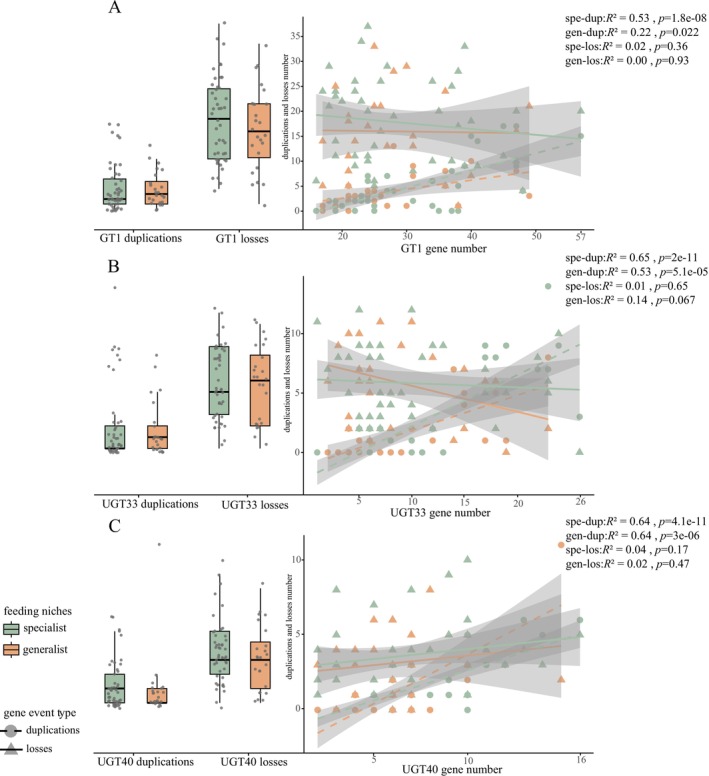
The correlation statistics for gene duplication and loss of *GT1* (A), *UGT33* (B), and *UGT40* (C) genes on terminal branches are presented in the figure. The left *Y*‐axis shows the number of gene duplications and losses in both generalist and specialist species. The right *Y*‐axis represents the correlation analysis between gene count and gene duplications/losses.

To test whether gene turnover was associated with host plant range, we examined the number of *GT1*, *UGT33*, and *UGT40* duplications and losses along terminal branches representing generalist and specialist species. We found no clear differences in *GT1* gene duplication or loss patterns between generalist and specialist butterflies (Figure 4A). Instead, rapid evolutionary dynamics characterized by frequent gene duplications and losses appear to be a general feature of *GT1* genes across all butterfly lineages. Correlation analysis between gene count and the *GT1*, *UGT33*, and *UGT40* genes across species reveals a significant positive correlation between gene duplication and gene number in both generalist and specialist species (Figure 4). However, no significant correlation was observed between gene loss and gene number in the terminal branches of species.

### 3.4 Tandem Duplication Dominates *GT1* Expansion With Subfamily‐Specific Patterns

**FIGURE 5:**
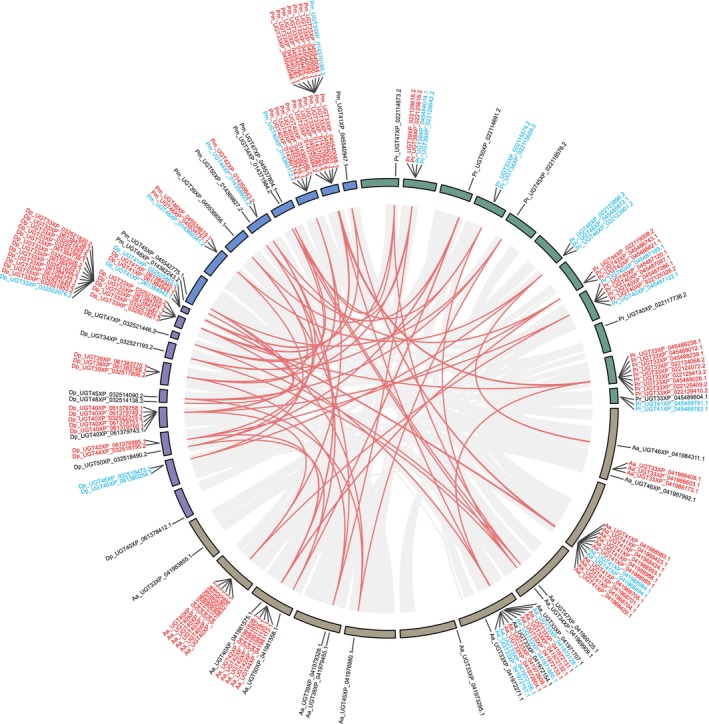
Genomic localization and duplication modes of identified *GT1* genes. Gene names consist of species abbreviations and gene IDs. The species include Aa (*Aricia agestis*), Dp ( *Danaus plexippus* ), Pm ( *Papilio machaon* ) and Pr ( *Pieris rapae* ). Gene colors represent duplication modes: red for tandem duplication, blue for proximal duplication, and black for dispersed duplication. Connecting lines indicate collinearity blocks between genomic segments, with red lines highlighting collinearity regions containing *GT1* genes.

In all four tested species, *GT1* genes were retained through gene duplication events. Among the identified duplication modes, tandem duplication (*A. agestis* [28 in 49], *D. plexippus* [29 in 41], *P. machaon* [21 in 31], *P. rapae* [18 in 34]) was the predominant mode contributing to *GT1* gene expansion (Figure 5). In contrast, *P. rapae* exhibited a relatively high proportion of *GT1* genes derived from proximal duplications. At the subfamily level, members of *UGT33* and *UGT40* predominantly underwent tandem duplications, whereas *UGT34*, *UGT39*, *UGT45*, *UGT47*, *UGT48*, and *UGT50* were primarily dispersed duplications. Notably, an expansion of *UGT41* genes was observed in *A. agestis*, with a greater number of *GT1* genes located outside collinearity regions. Overall, most *GT1* genes are located within collinearity blocks and originated through gene duplications.

### 3.5 Positive Selection Targets Functional Domains in Multiple *GT1* Subfamilies

To investigate the selective pressure on the *GT1* genes during evolution, we used the FEL and MEME models in HyPhy to detect positive selection at individual codon sites across *GT1* subfamilies. Based on these analyses, we identified significant signals of positive selection (*p* < 0.05) in the majority of *GT1* genes, suggesting that many members have undergone adaptive evolution (Table 1). In particular, multiple positively selected sites were detected in *UGT33* (5) and *UGT40* (7), indicating that these subfamilies have experienced functional selection. No positively selected sites were detected in *UGT34*, *UGT44*, or *UGT46*. Notably, positively selected sites were primarily located within the functional domains of *GT1* genes, with widespread distribution across both the N‐terminal substrate‐binding region and the C‐terminal donor‐binding region. *UGT33* primarily exhibited positive selection at the C‐terminal (659, 1562, 2185), while *UGT40* showed more positively selected amino acid sites at the N‐terminal (603, 753, 1401, 1403, 1428). This suggests that both functional domains may have been subject to selective pressures during evolution.

**TABLE 1:** The positions of positively selected sites within the region of different *UGT* genes.

	UGT domain N‐terminal	UGT domain C‐terminal	non‐domain region
*UGT33*	1276	659, 1562, 2185	37
*UGT39*			135
*UGT40*	603, 753, 1401, 1403, 1428	965	1212
*UGT41*	150		
*UGT42*		1353	
*UGT45*		1017	
*UGT47*		456	51
*UGT48*		479	
*UGT50*			19

### 3.6 Subfamily‐Specific Expression in Larval Gut and Adult Antenna

**FIGURE 6:**
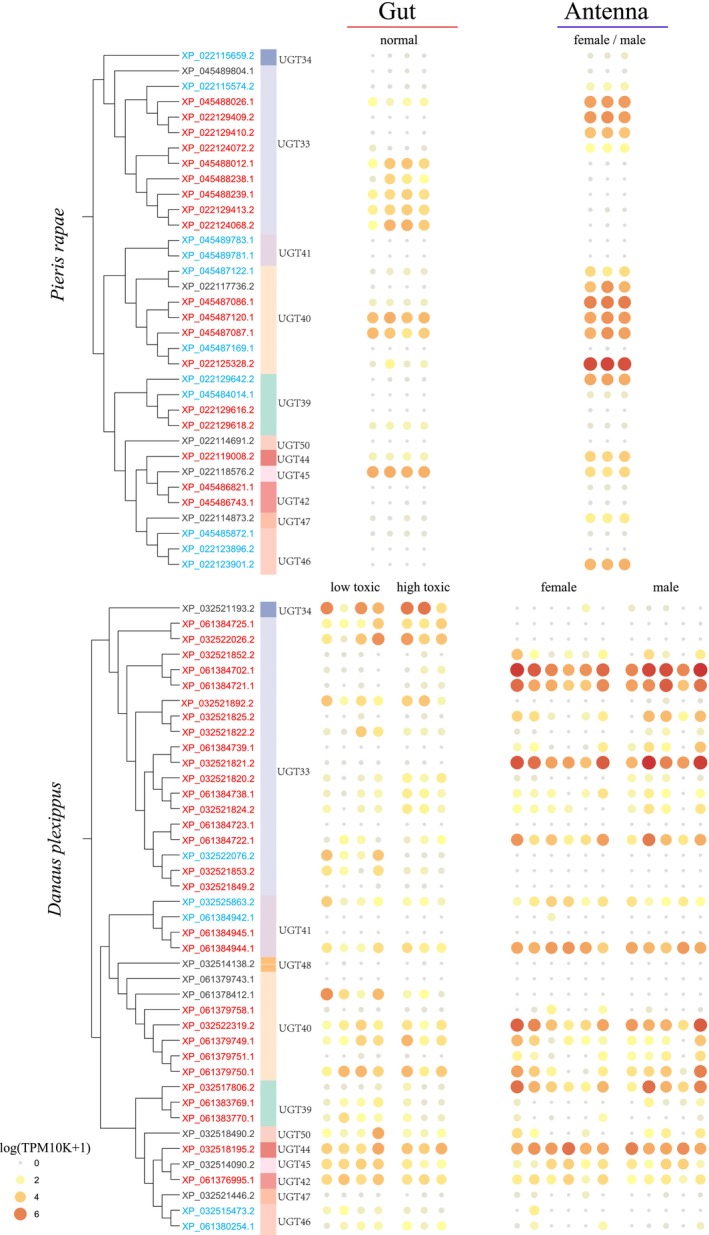
Expression profiles of *GT1* genes in the larval gut of the specialist species *Pieris rapae* and *Danaus plexippus* feeding on host plants with varying toxicity are presented. In addition, *GT1* gene expression in the antennae of adult butterflies of both species was compared. The color of each gene indicates its mode of duplication.

To further explore how different *GT1* genes are expressed in larval gut and adult antenna, the two main tissues associated with plant adaptation, we performed a combined analysis integrating gene expression data with *GT1* phylogenetic trees in *D. plexippus* and *P. rapae* . The overall expression of *GT1* genes showed a similar pattern in these two species, with genes highly expressed in adult antennae generally showing low expression in larval gut (Figure 6). *UGT44* and *UGT45* generally exhibited high expression levels, whereas *UGT41*, *UGT46*, and *UGT47* showed overall low expression in both tissues.

Within *UGT33* and *UGT40*, the two largest gene subfamilies, considerable variation in expression was observed among different genes. *UGT33s* (*XP_022124072.2*, *XP_022129410.2*, and *XP_022129409.2*) as well as *UGT40*s (*XP_022117736.2*, *XP_022125328.2*, *XP_022117736.2*, and *XP_045487122.1*) in *P. rapae* showed high expression in adult antennae but low expression in the larval gut. Similarly, *UGT33*s (*XP_061384721.1*, *XP_061384702.1*, *XP_061384722.1*, and *XP_032521821.2*) in *D. plexippus* were predominantly expressed in adult antennae. In contrast, other *UGT33s* in *P. rapae* (*XP_022124068.2*, *XP_022129413.2*, *XP_045488239.1*, *XP_045488238.1*, *XP_045488012.1*) and in *D. plexippus* (*XP_032522026.2*, *XP_061384725.1*, *XP_032521892.2*) exhibited high expression in the larval gut.

Interestingly, several genes closely located within the same phylogenetic branch exhibited markedly different expression patterns, such as *UGT40* genes in *P. rapae* (*XP_045487169.1* and *XP_022125328.2*), *UGT33* genes (*XP_032521821.2* and *XP_061384739.1*; *XP_061384722.1* and *XP_061384723.1*), and *UGT41* genes (*XP_061384944.1* and *XP_061384945.1*) in *D. plexippus* .

In contrast, some tandem duplicated genes maintained high expression in adult antennae, for example, *UGT33s* (*XP_045488239.1*, *XP_045488238.1*, and *XP_045488012.1*) and *UGT40* (*XP_022125328.2*) in *P. rapae* , and *UGT33* (*XP_061384721.1*; *XP_061384702.1*) in *D. plexippus* . In addition, certain genes, such as *UGT40s* in *P. rapae* (*XP_045487087.1* and *XP_045487120.1*) and in *D. plexippus* (*XP_061379750.1*, *XP_061379749.1*, *XP_032522319.2*), maintained high expression in both adult antennae and larval gut after tandem duplication.

## 4 Discussion

The interaction between butterflies and plants has been a focal point of evolutionary biology due to their coevolutionary processes, which involve the specialized metabolites in plants and the detoxification and plant‐feeding related enzymes in insects (Ehrlich and Raven 1964; Kawecki 1998; Janz and Nylin 1998). Among them, *GT1* genes play important roles in various pathways, including metabolite digestion and host selection, and consequently contribute significantly to the adaptation of insects to host plants. In this study, we systematically identified and analyzed the evolutionary patterns of the *GT1* gene family in butterflies, aiming to reveal its association with feeding habits, generalists and specialists of butterflies. The similar overall *GT1* gene numbers between generalist and specialist species suggest that butterflies shared a similar *GT1* repertoire for adaptation to host plant utilization. However, notable differences in the *GT1* copy numbers were observed between species as well as species within the same genus. This variation is not only reflected in the overall number of *GT1* genes but also in the *GT1* subfamilies of *UGT33* and *UGT44*, suggesting a complex evolutionary history of this family across butterfly species.

The *GT1* gene family in butterflies exhibits significant changes during evolution. Our analysis inferred a relatively high number of *GT1* gene copies in the common ancestor of butterflies (89), consistent with our previous studies and indicating an expanded *GT1* gene number in ancestral butterflies (Wu et al. 2024). Notably, both *UGT33* and *UGT40* genes exhibited large copy numbers in most butterfly species, indicating their functions in the adaptation of butterflies to plant feeding (Ahn et al. 2012). The *UGT33* and *UGT40* family members have been related to the adaptation of moths to different host diets with varied specialized chemicals (Israni et al. 2020; Wang et al. 2024). Further evidence revealed different expansion patterns of both subfamilies in butterfly families; several butterfly families, such as Nymphalidae, Lycaenidae, Riodinidae, and Pieridae, primarily had expanded *UGT33*, while others, including Papilionidae and Hesperiidae, had expanded *UGT40*. In addition, the considerable expansion of *UGT39* and *UGT41* in butterflies compared with other lepidopteran species indicates lineage‐specific gene duplications (Wang et al. 2024). These varied *GT1* subfamily expansions indicate diverse adaptive evolutionary traits of the *GT1* family in various butterfly lineages in response to ecological pressures, such as differences in host plant chemistry.

In contrast, several *UGT* subfamilies experienced a reduction in copy numbers during evolution. However, the number of gene losses in the terminal branches did not significantly correlate with the *GT1* gene number across species; instead, the duplication events in the terminal branches were positively correlated with the *GT1* copy numbers. This may be associated with small‐scale gene duplications and subsequent genomic rearrangements and deletions that occurred in the ancestral lepidopteran lineages and internal branches (Roelofs et al. 2020). The extensive small‐scale gene duplications in ancestral lepidopteran lineages, coupled with subsequent gene specialization and selective retention, likely contributed to the adaptive evolutionary arms race between butterflies and their host plants (Edger et al. 2015). Butterflies did not show a continuous expansion or contraction in their host ranges, but exhibited alternating appearances of special and general feeding, which reflect oscillations in the host utilization range (Janz et al. 2006; Ferrer‐Paris et al. 2013) with some lineages returning to the primitive host plants (Janz et al. 2006). These frequent host preference shifts likely led to the retention of some key *UGTs* during host adaptation and may also have driven the dynamic gene duplications and losses observed in *UGT* families as a molecular mechanism to cope with fluctuating host chemical defenses (Luo et al. 2025).

Most of the butterfly *GT1* genes were produced through tandem duplication in collinear regions, and this rapid amplification of gene copies likely promotes functional diversification (Zhou et al. 2019; Rogers et al. 2015), which may facilitate the adaptation of butterflies to different specialized chemicals related to shifted host plants. Recent work on the *UGTs* in the fall armyworm showed that the neofunctionalization of tandem duplications in the *UGT33* subfamily contributed to the varied toleration to host plant specialized chemicals (Luo et al. 2025). Furthermore, these tandemly duplicated genes support that a small number of duplications following gene losses in their ancestors shaped the *GT1* gene repertoire. Although the core function of *GT1* enzymes is to catalyze glycosyl transfer reactions in insects, several family members are also involved in various metabolic pathways by substrate diversity and tissue‐specific expression, including plant specialized metabolite transformation, odorant signal regulation, and detoxification responses (Ahmad et al. 1996; Ahn et al. 2012; He et al. 2017; Huang et al. 2008; Israni et al. 2020; Song et al. 2021).

The functional diversification or substrate diversity of *GT1s* in butterflies may be related to adaptive changes of specific sites in the GT1 enzymes. The selection pressure analyses showed at least one positively selected site in most subfamilies, suggesting that *GT1* genes had undergone adaptive evolution during the ecological diversification of butterfly species. The *UGT* domain typically consists of two functional regions, the N‐terminal region and the C‐terminal region. The N‐terminal region, responsible for substrate recognition and binding, exhibits high sequence variability among family members; the C‐terminal region, binding sugar donors such as UDP‐glucose, is generally conserved (Arriaza et al. 2023; Ahn et al. 2012). Using FEL and MEME models implemented in HyPhy, we identified several positively selected sites located within the functional domains, distributed across both the N‐terminal regions and C‐terminal regions. These results demonstrate that adaptive evolution in the *UGT* family involves not only diversification of substrate‐binding regions but also changes in donor‐binding regions (Arriaza et al. 2023). These positive selections may have driven functional divergence in both substrate and donor recognition capacities of certain *UGT* genes, contributing to host plant adaptation by binding diverse specialized metabolites or utilizing diverse sugar donors. In addition, mutations in a nonfunctional region can also contribute to the host range, as previous work on the fall armyworm showed the hindered related detoxification ability of host specialized chemicals and the reduced host width by a nonfunctional mutation (Wang et al. 2024). Furthermore, several *UGT* genes contain multiple *GT1* domains; the presence of multiple *UGT* domains in some genes may further increase the complexity of substrate recognition and functional regulation.

Gene duplication and subsequent changes in selective pressures may be coupled with tissue‐specific expression, facilitating the functional diversification of *GT1* genes (Innan and Kondrashov 2010; Gu et al. 2002; Genes 2000). Young duplicated genes often show tissue‐biased expression and gradually expand to other tissues in *Drosophila* (Jiang and Assis 2017; Assis and Bachtrog 2013); WGD‐derived gene duplications display more pronounced expression differences among tissues compared with single‐copy genes in *Arabidopsis* (Panchy et al. 2019; Wang et al. 2013). A similar pattern is observed in the *GT1* gene family. The *GT1* genes expressed in the larval gut mostly differ from those in adult antennae, indicating substantial functional divergence among *UGT* genes. This expression of *GT1* genes in the adult antennae and the larval gut likely reflects the different ecological roles of adult and larval stages in host utilization (Futuyma and Moreno 1988). For example, a *UGT33* highly expressed in *S. frugiperda* gut was primarily involved in larval detoxification capacity and adaptation to benzoxazinoids via glucosylation (Israni et al. 2020); while a *UGT46A6* highly expressed in *S. littoralis* antennae played a role in protecting the olfactory organs from xenobiotic compounds (Bozzolan et al. 2014). Notably, several *GT1* genes displayed generally high constitutive expression in the adult antennae, supporting their potential roles in host selection by chemical sensing or odorant perception (Bock 2016; Zhang et al. 2017). In addition, the high expression of a certain *GT1* gene in insect antennae is also related to sex recognition. A *UGT36* specifically expressed in *Drosophila melanogaster* antennae modulated sex pheromone discrimination and clearance (Fraichard et al. 2020). However, *UGT44* and *UGT45* exhibited high expression in both tissues, indicating their functions in both detoxification and sensory perception of plant secondary metabolites at different butterfly developmental stages.

*GT1* genes are broadly involved in detoxification processes in the gut, but only a few genes showed regulated expression under different feeding conditions (Tan et al. 2019). This is consistent with previous studies on host adaptation in butterflies, which showed a small number of *GT1* genes exhibiting changed expression when exposed to different plant specialized metabolites (Yu et al. 2016; Tan et al. 2019; Koenig et al. 2015). This suggests that the expression of limited rather than overall *GT1* genes was involved in butterfly adaptation to plant specialized metabolites through transcriptional changes. As their detoxification genes enable them to handle not only host plant specialized metabolites but also other specialized metabolites (Koenig et al. 2015), the detoxification capacity of plant specialized metabolites might not be the only limiting factor for butterflies in host selection.

Overall, the evolutionary changes of the *GT1* gene family, particularly the dynamic changes of gene copies and the positive selection in functional domains of different subfamilies, likely reflect their adaptation to diverse plant specialized metabolites in butterflies. Although no significant correlation was detected with host plant breadth, *GT1* copy numbers vary substantially across species. In addition, the divergent expression of *GT1* genes in the larval gut and adult antennae further supports that some *GT1* genes have undergone functional specialization to cope with plant‐derived chemical stressors. However, relatively conserved *GT1* gene expression patterns between the two representative species from two butterfly families indicate that *GT1* expression captures a limited spectrum of adaptations. Instead, positive selection of specific *GT1* genes in functional domains may be the main pathway facilitating adaptation to plant chemical defenses in butterflies.

However, this study has several limitations. Species were classified as generalists or specialists based on the number of host plant families. While this binary framework provides an initial comparative basis, the chemical profiles and morphological traits of host plants are similar across certain different families; consequently, generalist species may experience selective pressures comparable to those of specialists (Ali and Agrawal 2012). Furthermore, plants rarely rely on a single defense mechanism; multiple traits, such as chemical toxins, structural barriers, or nutrient limitation, are often employed simultaneously (Walling 2000). For instance, Poaceae not only produce benzoxazinoids as chemical deterrents but also develop silica‐based physical defenses (Massey et al. 2006; Wouters et al. 2014). Therefore, future work incorporating plant metabolite diversity and multitrait resistance strategies will be essential for fully elucidating the evolutionary dynamics of butterfly–host coadaptation.

## Author Contributions

**Jinyu Wu:** data curation (equal), formal analysis (equal), writing – original draft (equal), writing – review and editing (equal). **Hengyu Yan:** data curation (equal), formal analysis (equal). **Wanjiang Tang:** data curation (equal), formal analysis (equal). **Zhengyang Li:** data curation (equal), formal analysis (equal). **Amrita Chakraborty:** resources (equal), writing – review and editing (equal). **Zhengbo He:** resources (equal), writing – review and editing (equal). **Cao Zhou:** resources (equal), writing – review and editing (equal). **Shulin He:** resources (equal), writing – review and editing (equal).

## Conflicts of Interest

The authors declare no conflicts of interest.

## Supporting information


**Data S1:** ece372291‐sup‐0001‐Supinfo1.rar.

**Data S2:** ece372291‐sup‐0002‐Supinfo2.rar.

**Data S3:** ece372291‐sup‐0003‐TableS1.xlsx.

## Data Availability

All data used in this study were obtained from publicly available databases. The feeding habits of the species were obtained from previously published studies (Kawahara et al. 2023). The genome assemblies, their corresponding NCBI accession numbers, and the feeding habits of each species are listed in Table S1. The *GT1* gene sequences identified in this study are provided in *butt.faa*, and the known *UGT* reference sequences used for phylogenetic analysis are included in *otherseq.faa* (Data S1). The R script used for statistical analysis is available as *Statistical_Analysis. R* (Data S2).

## References

[ece372291-bib-0001] Ahmad, S. A. , T. L. Hopkins , and K. J. Kramer . 1996. “Tyrosine β‐Glucosyltransferase in the Tobacco Hornworm, *Manduca sexta* (L.): Properties, Tissue Localization, and Developmental Profile.” Insect Biochemistry and Molecular Biology 26, no. 1: 49–57.

[ece372291-bib-0002] Ahn, S.‐J. , H. Vogel , and D. G. Heckel . 2012. “Comparative Analysis of the UDP‐Glycosyltransferase Multigene Family in Insects.” Insect Biochemistry and Molecular Biology 42, no. 2: 133–147. 22155036 10.1016/j.ibmb.2011.11.006

[ece372291-bib-0003] Ali, J. G. , and A. A. Agrawal . 2012. “Specialist Versus Generalist Insect Herbivores and Plant Defense.” Trends in Plant Science 17: 293–302. 22425020 10.1016/j.tplants.2012.02.006

[ece372291-bib-0004] Allio, R. , B. Nabholz , S. Wanke , et al. 2021. “Genome‐Wide Macroevolutionary Signatures of Key Innovations in Butterflies Colonizing New Host Plants.” Nature Communications 12: 354. 10.1038/s41467-020-20507-3 PMC7806994 33441560

[ece372291-bib-0005] Arriaza, R. H. , B. Abiskaroon , M. Patel , et al. 2023. “Structural and Functional Studies Reveal the Molecular Basis of Substrate Promiscuity of a Glycosyltransferase Originating From a Major Agricultural Pest.” Journal of Biological Chemistry 299, no. 12: 105421. 37923139 10.1016/j.jbc.2023.105421 PMC10731231

[ece372291-bib-0006] Assis, R. , and D. Bachtrog . 2013. “Neofunctionalization of Young Duplicate Genes In Drosophila.” Proceedings of the National Academy of Sciences 110: 17409–17414. 10.1073/pnas.1313759110 PMC3808614 24101476

[ece372291-bib-0007] Bock, K. W. 2016. “The UDP‐Glycosyltransferase (UGT) Superfamily Expressed in Humans, Insects and Plants: Animal Plant Arms‐Race and Co‐Evolution.” Biochemical Pharmacology 99: 11–17. 26453144 10.1016/j.bcp.2015.10.001

[ece372291-bib-0008] Bozzolan, F. , D. Siaussat , A. Maria , et al. 2014. “Antennal Uridine Diphosphate (UDP)‐Glycosyltransferases in a Pest Insect: Diversity and Putative Function in Odorant and Xenobiotics Clearance.” Insect Molecular Biology 23: 539–549. 24698447 10.1111/imb.12100

[ece372291-bib-0009] Brady, D. , A. Saviane , S. Cappellozza , and F. Sandrelli . 2021. “The Circadian Clock in Lepidoptera.” Frontiers in Physiology 12: 776826. 34867483 10.3389/fphys.2021.776826 PMC8635995

[ece372291-bib-0010] Breeschoten, T. , C. F. H. van der Linden , D. R. Vera I , M. E. Schranz , and S. Simon . 2022. “Expanding the Menu: Are Polyphagy and Gene Family Expansions Linked across Lepidoptera?” Genome Biology and Evolution 14, no. 1: evab283. 34951642 10.1093/gbe/evab283 PMC8725640

[ece372291-bib-0011] Castro, É. 2018. “The Arms Race Between Heliconiine Butterflies and Passiflora Plants—New Insights on an Ancient Subject.” Biological Reviews 93: 555–573. 28901723 10.1111/brv.12357

[ece372291-bib-0012] Chen, C. , Y. Wu , J. Li , et al. 2023. “TBtools‐II: A “One for All, All for One” Bioinformatics Platform for Biological Big‐Data Mining.” Molecular Plant 16, no. 11: 1733–1742. 37740491 10.1016/j.molp.2023.09.010

[ece372291-bib-0013] Chen, S. 2023. “Ultrafast one‐Pass FASTQ Data Preprocessing, Quality Control, and Deduplication Using Fastp.” iMeta 2, no. 2: e107. 38868435 10.1002/imt2.107 PMC10989850

[ece372291-bib-0014] Danecek, P. , J. K. Bonfield , J. Liddle , et al. 2021. “Twelve Years of SAMtools and BCFtools.” GigaScience 10, no. 2: giab008. 33590861 10.1093/gigascience/giab008 PMC7931819

[ece372291-bib-0015] Després, L. , J.‐P. David , and C. Gallet . 2007. “The Evolutionary Ecology of Insect Resistance to Plant Chemicals.” Trends in Ecology & Evolution 22: 298–307. 17324485 10.1016/j.tree.2007.02.010

[ece372291-bib-0016] Drost, H.‐G. , A. Gabel , I. Grosse , and M. Quint . 2015. “Evidence for Active Maintenance of Phylotranscriptomic Hourglass Patterns in Animal and Plant Embryogenesis.” Molecular Biology and Evolution 32, no. 5: 1221–1231. 25631928 10.1093/molbev/msv012 PMC4408408

[ece372291-bib-0017] Durand, D. , B. V. Halldórsson , and B. Vernot . 2006. “A Hybrid Micro‐Macroevolutionary Approach to Gene Tree Reconstruction.” Journal of Computational Biology 13, no. 2: 320–335. 16597243 10.1089/cmb.2006.13.320

[ece372291-bib-0018] Edgar, R. C. 2004. “MUSCLE: Multiple Sequence Alignment With High Accuracy and High Throughput.” Nucleic Acids Research 32, no. 5: 1792–1797. 15034147 10.1093/nar/gkh340 PMC390337

[ece372291-bib-0019] Edger, P. P. , H. M. Heidel‐Fischer , M. Bekaert , et al. 2015. “The Butterfly Plant Arms‐Race Escalated by Gene and Genome Duplications.” Proceedings of the National Academy of Sciences 112: 8362–8366. 10.1073/pnas.1503926112 PMC4500235 26100883

[ece372291-bib-0020] Ehrlich, P. R. , and P. H. Raven . 1964. “Butterflies and Plants: A Study in Coevolution.” Evolution 18: 586–608.

[ece372291-bib-0022] Ferrer‐Paris, J. R. , A. Sánchez‐Mercado , Á. L. Viloria , and J. Donaldson . 2013. “Congruence and Diversity of Butterfly‐Host Plant Associations at Higher Taxonomic Levels.” PLoS One 8, no. 5: e63570. 23717448 10.1371/journal.pone.0063570 PMC3662771

[ece372291-bib-0023] Fraichard, S. , A. Legendre , P. Lucas , et al. 2020. “Modulation of Sex Pheromone Discrimination by a UDP‐Glycosyltransferase in Drosophila Melanogaster.” Genes 11: 237. 32106439 10.3390/genes11030237 PMC7140800

[ece372291-bib-0024] Futuyma, D. J. , and G. Moreno . 1988. “The Evolution of Ecological Specialization.” Annual Review of Ecology and Systematics 19: 207–233.

[ece372291-bib-0025] Gallon, M. E. , and A. M. Smilanich . 2023. “Effects of Host Plants on Development and Immunity of a Generalist Insect Herbivore.” Journal of Chemical Ecology 49: 142–154. 36763248 10.1007/s10886-023-01410-9

[ece372291-bib-0026] Genes, D. 2000. “The Evolutionary Fate and Consequences of.” Science 290: 1151. 11073452 10.1126/science.290.5494.1151

[ece372291-bib-0027] Greenstein, L. , C. Steele , and C. M. Taylor . 2022. “Host Plant Specificity of the Monarch Butterfly *Danaus plexippus*: A Systematic Review and Meta‐Analysis.” PLoS One 17: e0269701. 35700160 10.1371/journal.pone.0269701 PMC9197062

[ece372291-bib-0028] Gu, Z. , D. Nicolae , H. H. Lu , and W.‐H. Li . 2002. “Rapid Divergence in Expression Between Duplicate Genes Inferred From Microarray Data.” Trends in Genetics 18: 609–613. 12446139 10.1016/s0168-9525(02)02837-8

[ece372291-bib-0029] Haribal, M. , and J. Renwick . 1998. “Identification and Distribution of Oviposition Stimulants for Monarch Butterflies in Hosts and Nonhosts.” Journal of Chemical Ecology 24: 891–904.

[ece372291-bib-0030] Haribal, M. , and J. A. A. Renwick . 1996. “Oviposition Stimulants for the Monarch Butterfly: Flavonol Glycosides From *Asclepias curassavica* .” Phytochemistry 41: 139–144. 8588865 10.1016/0031-9422(95)00511-0

[ece372291-bib-0031] He, P. , Y.‐F. Zhang , D.‐Y. Hong , et al. 2017. “A Reference Gene Set for Sex Pheromone Biosynthesis and Degradation Genes From the *Diamondback moth*, *Plutella xylostella*, Based on Genome and Transcriptome Digital Gene Expression Analyses.” BMC Genomics 18: 1–19. 28249567 10.1186/s12864-017-3592-y PMC5333385

[ece372291-bib-0032] Huang, F.‐F. , C.‐L. Chai , Z. Zhang , et al. 2008. “The UDP‐Glucosyltransferase Multigene Family in *Bombyx mori* .” BMC Genomics 9: 1–14. 19038024 10.1186/1471-2164-9-563 PMC2633020

[ece372291-bib-0033] Innan, H. , and F. Kondrashov . 2010. “The Evolution of Gene Duplications: Classifying and Distinguishing Between Models.” Nature Reviews Genetics 11: 97–108. 10.1038/nrg2689 20051986

[ece372291-bib-0034] Inoue, T. A. , M. Suetake , N. Nishidzu , F. Yokohari , K. Niihara , and T. Fukuda . 2023. “Behavioral and Electrophysiological Study on Eight Japanese Papilio Species With Five Hostplant Volatiles and Linalool.” Journal of Chemical Ecology 49: 397–407. 37378686 10.1007/s10886-023-01433-2 PMC10611675

[ece372291-bib-0035] Israni, B. , F. C. Wouters , K. Luck , et al. 2020. “The Fall Armyworm *Spodoptera frugiperda* Utilizes Specific UDP‐Glycosyltransferases to Inactivate Maize Defensive Benzoxazinoids.” Frontiers in Physiology 11: 604754. 33408643 10.3389/fphys.2020.604754 PMC7781194

[ece372291-bib-0036] Janz, N. , and S. Nylin . 1998. “Butterflies and Plants: A Phylogenetic Study.” Evolution 52, no. 2: 486–502. 28568350 10.1111/j.1558-5646.1998.tb01648.x

[ece372291-bib-0037] Janz, N. , S. Nylin , and N. Wahlberg . 2006. “Diversity Begets Diversity: Host Expansions and the Diversification of Plant‐Feeding Insects.” BMC Evolutionary Biology 6: 1–10. 16420707 10.1186/1471-2148-6-4 PMC1382262

[ece372291-bib-0038] Jiang, X. , and R. Assis . 2017. “Natural Selection Drives Rapid Functional Evolution of Young Drosophila Duplicate Genes.” Molecular Biology and Evolution 34: 3089–3098. 28961791 10.1093/molbev/msx230 PMC5850746

[ece372291-bib-0039] Katoh, K. , and D. M. Standley . 2013. “MAFFT Multiple Sequence Alignment Software Version 7: Improvements in Performance and Usability.” Molecular Biology and Evolution 30, no. 4: 772–780. 23329690 10.1093/molbev/mst010 PMC3603318

[ece372291-bib-0040] Kawahara, A. Y. , C. Storer , A. P. S. Carvalho , et al. 2023. “A Global Phylogeny of Butterflies Reveals Their Evolutionary History, Ancestral Hosts and Biogeographic Origins.” Nature Ecology & Evolution 7, no. 6: 903–913. 37188966 10.1038/s41559-023-02041-9 PMC10250192

[ece372291-bib-0041] Kawecki, T. J. 1998. “Red Queen Meets *Santa rosalia*: Arms Races and the Evolution of Host Specialization in Organisms With Parasitic Lifestyles.” American Naturalist 152, no. 4: 635–651. 10.1086/286195 18811369

[ece372291-bib-0042] Kim, D. , J. M. Paggi , C. Park , C. Bennett , and S. L. Salzberg . 2019. “Graph‐Based Genome Alignment and Genotyping With HISAT2 and HISAT‐Genotype.” Nature Biotechnology 37, no. 8: 907–915. 10.1038/s41587-019-0201-4 PMC7605509 31375807

[ece372291-bib-0043] Koenig, C. , A. Bretschneider , D. G. Heckel , E. Grosse‐Wilde , B. S. Hansson , and H. Vogel . 2015. “The Plastic Response of *Manduca sexta* to Host and Non‐Host Plants.” Insect Biochemistry and Molecular Biology 63: 72–85. 26070471 10.1016/j.ibmb.2015.06.001

[ece372291-bib-0045] Li, W. , and A. Godzik . 2006. “Cd‐Hit: A Fast Program for Clustering and Comparing Large Sets of Protein or Nucleotide Sequences.” Bioinformatics 22, no. 13: 1658–1659. 16731699 10.1093/bioinformatics/btl158

[ece372291-bib-0046] Liao, Y. , G. K. Smyth , and W. Shi . 2014. “featureCounts: An Efficient General Purpose Program for Assigning Sequence Reads to Genomic Features.” Bioinformatics 30, no. 7: 923–930. 24227677 10.1093/bioinformatics/btt656

[ece372291-bib-0047] Luo, M. , B. Li , L. Ma , et al. 2025. “Multiple Evolutionary Events in Host Plant Adaptation in Lepidoptera.” *bioRxiv*. 2025.04. 05.647383. 10.1111/pce.70502 41906325

[ece372291-bib-0048] Massey, F. P. , A. R. Ennos , and S. Hartley . 2006. “Silica in Grasses as a Defence Against Insect Herbivores: Contrasting Effects on Folivores and a Phloem Feeder.” Ecology 75: 595–603. 10.1111/j.1365-2656.2006.01082.x 16638012

[ece372291-bib-0049] Munro, C. , F. Zapata , M. Howison , S. Siebert , and C. W. Dunn . 2022. “Evolution of Gene Expression Across Species and Specialized Zooids in Siphonophora.” Molecular Biology and Evolution 39: msac027. 35134205 10.1093/molbev/msac027 PMC8844502

[ece372291-bib-0050] Murrell, B. , J. O. Wertheim , S. Moola , et al. 2012. “Detecting Individual Sites Subject to Episodic Diversifying Selection.” PLoS Genetics 8, no. 7: e1002764. 22807683 10.1371/journal.pgen.1002764 PMC3395634

[ece372291-bib-0052] Nagare, M. , M. Ayachit , A. Agnihotri , W. Schwab , and R. Joshi . 2021. “Glycosyltransferases: The Multifaceted Enzymatic Regulator in Insects.” Insect Molecular Biology 30: 123–137. 33263941 10.1111/imb.12686

[ece372291-bib-0085] Nallu, S. , J. A. Hill , K. Don , et al. 2018. “The Molecular Genetic Basis of Berbivory Between Butterflies and Their Host Plants.” Nature Ecology & Evolution 2, no. 9: 1418–1427. 30076351 10.1038/s41559-018-0629-9 PMC6149523

[ece372291-bib-0053] Nguyen, L. T. , H. A. Schmidt , A. von Haeseler , and B. Q. Minh . 2015. “IQ‐TREE: A Fast and Effective Stochastic Algorithm for Estimating Maximum‐Likelihood Phylogenies.” Molecular Biology and Evolution 32, no. 1: 268–274. 25371430 10.1093/molbev/msu300 PMC4271533

[ece372291-bib-0054] Nishida, R. 2002. “Sequestration of Defensive Substances From Plants by Lepidoptera.” Annual Review of Entomology 47: 57–92. 10.1146/annurev.ento.47.091201.145121 11729069

[ece372291-bib-0055] Panchy, N. L. , C. B. Azodi , E. F. Winship , et al. 2019. “Expression and Regulatory Asymmetry of Retained *Arabidopsis thaliana* Transcription Factor Genes Derived From Whole Genome Duplication.” BMC Evolutionary Biology 19: 77. 30866803 10.1186/s12862-019-1398-z PMC6416927

[ece372291-bib-0056] Paradis, E. , and K. Schliep . 2019. “ape 5.0: An Environment for Modern Phylogenetics and Evolutionary Analyses in R.” Bioinformatics 35, no. 3: 526–528. 30016406 10.1093/bioinformatics/bty633

[ece372291-bib-0057] Pertea, G. , and M. Pertea . 2020. “GFF Utilities: GffRead and GffCompare.” F1000Research 9: ISCB‐Comm. 10.12688/f1000research.23297.1 PMC7222033 32489650

[ece372291-bib-0058] Pond, K. , and S. D. Frost . 2005. “Not so Different After All: A Comparison of Methods for Detecting Amino Acid Sites Under Selection.” Molecular Biology and Evolution 22, no. 5: 1208–1222. 15703242 10.1093/molbev/msi105

[ece372291-bib-0059] Rane, R. V. , T. K. Walsh , S. L. Pearce , et al. 2016. “Are Feeding Preferences and Insecticide Resistance Associated With the Size of Detoxifying Enzyme Families in Insect Herbivores?” Current Opinion in Insect Science 13: 70–76. 27436555 10.1016/j.cois.2015.12.001

[ece372291-bib-0060] Renwick, J. , and K. Lopez . 1999. “Experience‐Based Food Consumption by Larvae of *Pieris rapae*: Addiction to Glucosinolates?” Entomologia Experimentalis et Applicata 91: 51–58.

[ece372291-bib-0061] Reudler Talsma, J. H. , A. Biere , J. A. Harvey , and S. van Nouhuys . 2008. “Oviposition Cues for a Specialist Butterfly—Plant Chemistry and Size.” Journal of Chemical Ecology 34: 1202–1212. 18612691 10.1007/s10886-008-9519-y PMC2518948

[ece372291-bib-0062] Richards, L. A. , E. C. Lampert , M. D. Bowers , C. D. Dodson , A. M. Smilanich , and L. A. Dyer . 2012. “Synergistic Effects of Iridoid Glycosides on the Survival, Development and Immune Response of a Specialist Caterpillar, *Junonia coenia* (Nymphalidae).” Journal of Chemical Ecology 38: 1276–1284. 23053916 10.1007/s10886-012-0190-y

[ece372291-bib-0063] Roelofs, D. , A. Zwaenepoel , T. Sistermans , et al. 2020. “Multi‐Faceted Analysis Provides Little Evidence for Recurrent Whole‐Genome Duplications During Hexapod Evolution.” BMC Biology 18: 1–13. 32460826 10.1186/s12915-020-00789-1 PMC7251882

[ece372291-bib-0064] Rogers, R. L. , J. M. Cridland , L. Shao , T. T. Hu , P. Andolfatto , and K. R. Thornton . 2015. “Tandem Duplications and the Limits of Natural Selection in *Drosophila yakuba* and *Drosophila simulans* .” PLoS One 10, no. 7: e0132184. 26176952 10.1371/journal.pone.0132184 PMC4503668

[ece372291-bib-0065] Rohlfs, R. V. , and R. Nielsen . 2015. “Phylogenetic ANOVA: The Expression Variance and Evolution Model for Quantitative Trait Evolution.” Systematic Biology 64, no. 5: 695–708. 26169525 10.1093/sysbio/syv042 PMC4635652

[ece372291-bib-0066] Song, W. , Y. Fan , F. Zhu , R. H. Taha , and K. Chen . 2021. “The Expression of UGT46A1 Gene and Its Effect on Silkworm Feeding.” Processes 9, no. 8: 1473.

[ece372291-bib-0067] Suyama, M. , D. Torrents , and P. Bork . 2006. “PAL2NAL: Robust Conversion of Protein Sequence Alignments Into the Corresponding Codon Alignments.” Nucleic Acids Research 34: W609–W612. 16845082 10.1093/nar/gkl315 PMC1538804

[ece372291-bib-0068] Tan, W. H. , T. Acevedo , E. V. Harris , et al. 2019. “Transcriptomics of Monarch Butterflies (*Danaus plexippus*) Reveals that Toxic Host Plants Alter Expression of Detoxification Genes and Down‐Regulate a Small Number of Immune Genes.” Molecular Ecology 28, no. 22: 4845–4863. 31483077 10.1111/mec.15219

[ece372291-bib-0069] Teze, D. , G. N. Bidart , and D. H. Welner . 2022. “Family 1 Glycosyltransferases (GT1, UGTs) Are Subject to Dilution‐Induced Inactivation and Low Chemo Stability Toward Their Own Acceptor Substrates.” Frontiers in Molecular Biosciences 9: 909659. 35936788 10.3389/fmolb.2022.909659 PMC9354691

[ece372291-bib-0070] Thompson, J. D. , J.‐C. Thierry , and O. Poch . 2003. “RASCAL: Rapid Scanning and Correction of Multiple Sequence Alignments.” Bioinformatics 19, no. 9: 1155–1161. 12801878 10.1093/bioinformatics/btg133

[ece372291-bib-0071] van der Linden, C. F. H. , M. F. Wallisdevries , and S. Simon . 2021. “Great Chemistry Between Us: The Link Between Plant Chemical Defenses and Butterfly Evolution.” Ecology and Evolution 11: 8595–8613. 34257918 10.1002/ece3.7673 PMC8258229

[ece372291-bib-0072] Walling, L. L. 2000. “The Myriad Plant Responses to Herbivores.” Journal of Plant Growth Regulation 19: 195–216. 11038228 10.1007/s003440000026

[ece372291-bib-0073] Wang, H. , J. Song , B. J. Hunt , et al. 2024. “UDP‐Glycosyltransferases Act as Key Determinants of Host Plant Range in Generalist and Specialist Spodoptera Species.” Proceedings of the National Academy of Sciences 121, no. 19: e2402045121. 10.1073/pnas.2402045121 PMC11087754 38683998

[ece372291-bib-0074] Wang, Y. , X. Tan , and A. H. Paterson . 2013. “Different Patterns of Gene Structure Divergence Following Gene Duplication in Arabidopsis.” BMC Genomics 14: 652. 24063813 10.1186/1471-2164-14-652 PMC3848917

[ece372291-bib-0075] Wang, Y. , H. Tang , J. D. Debarry , et al. 2012. “MCScanX: A Toolkit for Detection and Evolutionary Analysis of Gene Synteny and Collinearity.” Nucleic Acids Research 40, no. 7: e49. 22217600 10.1093/nar/gkr1293 PMC3326336

[ece372291-bib-0076] Waterhouse, R. M. , M. Seppey , F. A. Simão , et al. 2018. “BUSCO Applications From Quality Assessments to Gene Prediction and Phylogenomics.” Molecular Biology and Evolution 35, no. 3: 543–548. 29220515 10.1093/molbev/msx319 PMC5850278

[ece372291-bib-0077] Wouters, F. C. , M. Reichelt , G. Glauser , et al. 2014. “Reglucosylation of the Benzoxazinoid DIMBOA With Inversion of Stereochemical Configuration Is a Detoxification Strategy in Lepidopteran Herbivores.” Angewandte Chemie International Edition 53: 11320–11324. 25196135 10.1002/anie.201406643

[ece372291-bib-0078] Wu, J. , W. Tang , Z. Li , et al. 2024. “Duplications and Losses of the Detoxification Enzyme Glycosyltransferase 1 Are Related to Insect Adaptations to Plant Feeding.” International Journal of Molecular Sciences 25, no. 11: 6080. 38892266 10.3390/ijms25116080 PMC11173166

[ece372291-bib-0079] Yonekura‐Sakakibara, K. , and K. Hanada . 2011. “An Evolutionary View of Functional Diversity in Family 1 Glycosyltransferases.” Plant Journal 66, no. 1: 182–193. 10.1111/j.1365-313X.2011.04493.x 21443631

[ece372291-bib-0080] Yu, Q. Y. , S. M. Fang , Z. Zhang , and C. D. Jiggins . 2016. “The Transcriptome Response of *Heliconius melpomene* Larvae to a Novel Host Plant.” Molecular Ecology 25, no. 19: 4850–4865. 27572947 10.1111/mec.13826

[ece372291-bib-0082] Zhang, Y.‐N. , J.‐F. Ma , L. Xu , et al. 2017. “Identification and Expression Patterns of UDP‐Glycosyltransferase (UGT) Genes From Insect Pest *Athetis lepigone* (Lepidoptera: Noctuidae).” Journal of Asia‐Pacific Entomology 20, no. 1: 253–259.

[ece372291-bib-0083] Zheng, J. , Q. Ge , Y. Yan , X. Zhang , L. Huang , and Y. Yin . 2023. “dbCAN3: Automated Carbohydrate‐Active Enzyme and Substrate Annotation.” Nucleic Acids Research 51, no. W1: W115–W121. 37125649 10.1093/nar/gkad328 PMC10320055

[ece372291-bib-0084] Zhou, Y. , X. Li , S. Katsuma , et al. 2019. “Duplication and Diversification of Trehalase Confers Evolutionary Advantages on Lepidopteran Insects.” Molecular Ecology 28, no. 24: 5282–5298. 31674075 10.1111/mec.15291

